# The influence of paediatric HIV infection on circulating B cell subsets and CXCR5^+^ T helper cells

**DOI:** 10.1111/cei.12618

**Published:** 2015-05-06

**Authors:** A. Bamford, M. Hart, H. Lyall, D. Goldblatt, P. Kelleher, B. Kampmann

**Affiliations:** ^1^ Section of Paediatrics Division of Infectious Diseases; ^2^ Section of Immunology Division of Infectious Diseases Imperial College; ^3^ Department of Paediatric Infectious Diseases Imperial College Healthcare NHS Trust; ^4^ Immunobiology Unit Institute of Child Health, University College London London UK; ^5^ MRC Unit, The Gambia Vaccinology Theme, Fajara The Gambia West Africa

**Keywords:** B cell, follicular T helper, HIV, memory, paediatric

## Abstract

Antiretroviral therapy (ART) only partially restores HIV‐induced alterations in lymphocyte populations. We assessed B and T cell phenotypes in a cohort of children from a single centre in the United Kingdom with perinatally acquired HIV compared to healthy controls. The majority of HIV infected children (44 of 56) were on fully suppressive combination ART. Children with perinatally acquired HIV had significantly lower memory B and CD4^+^CD45RO^+^CXCR5^+^ [follicular T helper cell (Tfh)‐like] T cell percentages. Detectable viraemia was associated with higher CD21^–^ (activated and exhausted/tissue‐like memory) B cells. A greater proportion of life spent on suppressive ART was associated with higher memory B cell percentages. These results suggest that early and sustained suppressive ART may preserve B and T cell phenotypes in perinatally acquired HIV and limit deficits in humoral immunity. A lower proportion of circulating Tfh‐like cells in HIV infected children appears to be independent of HIV treatment history and ongoing HIV viraemia and warrants further investigation.

## Introduction

Typically perceived as causing an immune defect affecting T cells, there are an expanding number of reports describing B cell phenotype in the context of perinatally acquired HIV. It is now clear that HIV infection is associated with phenotypical abnormalities of B cells both on and off antiretroviral therapy (ART). How ART treatment history impacts upon potential for B cell immune preservation/reconstitution in perinatally acquired HIV has evolved more recently as a topic of interest in children 1, 2, 3, 4.

More is known about B cell phenotypical changes in HIV‐infected adults. Observed abnormalities can be partially reversed by ART, while earlier initiation of ART can result in the preservation of B cell phenotype and function (reviewed in 5). There are, of course, important differences in the immunological characteristics of perinatally acquired HIV, and adult infection and caution must be taken when extrapolating from adult data to paediatric populations: perinatally acquired HIV results in an insult to a naive and still‐developing immune system, which is reflected in differences in the dynamics of viral replication, immunosuppression, clinical progression and response to ART 6.

There is a paucity of data concerning circulating CD4^+^CD45RO^+^CXCR5^+^ (follicular T helper (Tfh)‐like) cells in children and no published studies in perinatally acquired HIV. Debate continues regarding how best to define Tfh‐like cells and how they relate to germinal centre Tfh. The heterogeneity of studies relating to cell surface markers, cytokines analysed and cell co‐culture methods have so far limited our understanding of how this subset may be best characterized, together with their developmental stage in relation to antigen exposure. However, current evidence suggests that circulating Tfh‐like cells are linked closely to Tfh, already known to be key players in the B cell response to T‐dependent antigens and resultant B cell differentiation 5, 7, 8, 9. The importance of Tfh in HIV infection is increasingly acknowledged. Tfh populations have been shown to be particularly susceptible to HIV infection and are expanded in secondary lymphoid tissue in non‐human primates with simian immunodeficiency virus (SIV) infection and in humans with chronic HIV infection. Furthermore, this correlates with expansion of germinal centre B cell populations. ART is associated with partial normalization of these observed differences (reviewed in 5 and 7).

The aims of this study were to determine the influence of paediatric HIV infection on circulating Tfh‐like cells and to assess if there was an association between Tfh‐like cells and memory B cell subsets. By assessing the relationship between cell phenotype and current and past ART status, we highlight the potential for early and sustained ART to preserve normal lymphocyte (Tfh/B cell) development.

## Methods

Children with perinatally acquired HIV (HIV^+^) and healthy, HIV uninfected children (HIV^−^) aged 2 months–18 years were recruited from the HIV family clinic and preoperative assessment clinics at Imperial College Healthcare NHS Trust, St Mary's Hospital, London (August 2009–October 2012). All eligible HIV^+^ children were approached. Those consenting to take part in the study were included. Ethical approval was obtained from Riverside Research Ethics Committee, Charing Cross Hospital, London (reference: 09/H0706/23). The parents of children included in the study provided written informed consent and human experimentation guidelines for Imperial College London were followed. Background demographic and clinical information were obtained from clinical notes, general practitioner, hospital pathology and personal child health records. Exclusion criteria included ongoing febrile illness, pregnancy, additional cause of immunosuppression, co‐infection with tuberculosis, hepatitis B, hepatitis C and HIV‐related malignancy. Ongoing HIV viraemia in the context of known resistance to current anti‐retroviral regimen was not an exclusion criterion.

**Figure 1:**
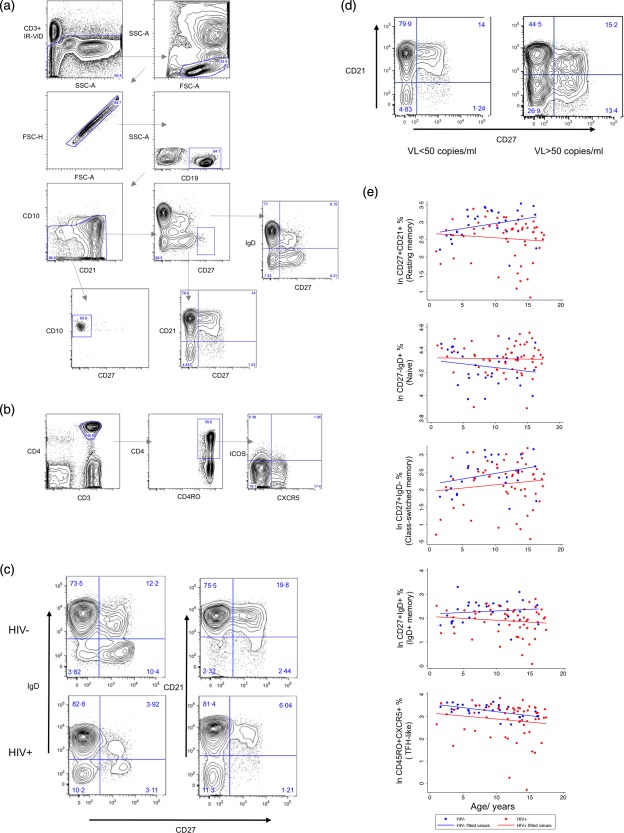
Gating strategy for identification of (a) B cell subsets and (b) T cell subsets. Following identification of live CD3^−^ cells, CD19^+^ B lymphocytes were identified by sequentially gating forward‐scatter (FSC)‐A *versus* side‐scatter (SSC)‐A, FSC‐A *versus* FSC‐H, CD19 *versus* SSC‐A. CD10^+^CD27^−^ and CD27^++^ immunoglobulin (Ig)D^−^ cells were then quantified. The remaining cells were subdivided in two ways: CD27 *versus* IgD and CD27 *versus* CD21, resulting in eight further subsets. Following identification of live CD19^−^CD14^−^ cells, CD4^+^ T lymphocytes were identified by sequentially gating FSC‐A *versus* SSC‐A, FSC‐A *versus* FSC‐H, CD3 *versus* CD4. Further subsets were identified using CD45RO, CXCR5 and inducible T cell co‐stimulator (ICOS). Typical contour plots from (c) a healthy child and a child with perinatally acquired HIV of approximately the same age, (d) a child with detectable viral load (VL) and a child with VL < 50 copies/ml. Children with perinatally acquired HIV have higher percentages of naive B cell subsets (CD27^−^) and a correspondingly lower percentage of memory subsets (CD27^+^) compared to healthy children. Detectable VL is associated with an over‐representation of CD21^−^ populations (CD27^+^CD21^−^ and CD27^−^CD21^−^). (e) Regression plots comparing healthy children with children with perinatally acquired HIV. Subsets are reported as described for Table 1. When comparing HIV^−^ with HIV^+^ groups, significant differences in CD27^+^CD21^+^, CD27^−^IgD^+^, CD27^+^IgDv, CD27^+^IgD^−^ and CD45RO^+^CXCR5^+^ cells were observed after adjusting for age (*P < *0·05) HIV^− ^= HIV uninfected child healthy control; HIV^+^ = HIV‐infected child; VL= HIV viral load; ln = natural log.

HIV viral load (VL) was measured using branched‐chain DNA assay (Siemens Healthcare, Erlangen, Germany) [lower limit of detection 50 copies/ml (c/ml)]. Lymphocyte subsets (LSS) were measured by staining whole blood [ethylenediamine tetraacetic acid (EDTA)] with monoclonal antibodies [CD45‐fluorescein isothiocyanate (FITC), CD4‐RD1, CD8‐phycoerythrin‐Texas red (ECD), CD3‐PC5, CD56‐RD1, CD19‐ECD, CD16‐phycoerythrin (PE) (Beckman Coulter, Brea, CA, USA] and evaluating using FC500 flow cytometer (Beckman Coulter). T and B cell subsets were identified from whole blood using six‐colour flow cytometry: CD3^+^CD4^+^CD45RO^+^ (memory T), CD3^+^CD4^+^CD45RO^+^CXCR5^+^ (Tfh‐like), CD3^+^CD4^+^CD45RO^+^CXCR5^+^ inducible T cell co‐stimulator (ICOS)^+^ (ICOS^+^Tfh‐like), CD19^+^CD10^+^CD21^lo^CD27^−^ (transitional B), CD19^+^CD10^−^immunoglobulin (Ig) D^−^CD27^++^ (plasmablasts), CD19^+^CD10^−^CD27^−^IgD^+^ (naive B), CD19^+^CD10^−^CD27^+^IgD^+^ (IgD^+^ memory B), CD19^+^CD10^−^CD27^+^IgD^–^ (class‐switched memory B), CD19^+^CD10^−^CD27^−^IgD^−^ (double‐negative B), CD19^+^CD10^–^CD27^–^CD21^+^ (naive mature B), CD19^+^CD10^–^CD27^+^CD21^+^ (resting memory B), CD19^+^CD10^−^CD27^+^CD21^−^ (activated memory B), CD19^+^CD10^−^CD27^−^CD21^−^ (exhausted/tissue‐like memory B). Subsets were quantified by staining with live/dead amine reactive dye (Invitrogen, Carlsbad, CA, USA) and monoclonal antibodies: IgD‐FITC, CD19‐PE‐cyanin 7 (Cy7) (Beckman Coulter), CD27‐allophyocyanin (APC), CD21‐PE, CD3‐APC‐Cy7 (BD Biosciences, San Jose, CA, USA), CD10‐peridinin chlorophyll (PerCP) (Exbio, Vestec, Czech Republic) for B cell subsets and CD3‐APC‐Cy7, CXCR5‐Axf647, ICOS‐PE, CD45RO‐FITC (BD Biosciences) and CD4‐PE‐Cy7, CD14‐ECD, CD19‐ECD (Beckman Coulter) for T cell subsets. Acquisition was performed on an LSR II (BD Biosciences). A minimum of 10 000 CD19 events and 30 000 CD4 events were acquired. Data were analysed using FlowJo software version 9.4 (TreeStar Inc., Ashland, OR, USA). To allow comparison with currently available literature, gating strategy was guided by existing reports on memory B cell and Tfh‐like cell phenotype 10, 11, 12 and is shown in Fig. 1a,b.

Analysis was performed using Stata IC version 12·1 for Mac (StataCorp, College Station, TX, USA). χ^2^ and Fisher's exact test were used to compare proportions and the Mann–Whitney *U*‐test to compare baseline age and linear regression was used to compare log‐transformed cell subset data between groups (allowing adjustment for age and to assess interactions between age and group). Linear regression was also used to investigate the relationship between lymphocyte, B and T cell subsets and clinical history and the association between T and B cell subsets within the HIV^+^ group. Clinical variables assessed included viral load > 50 c/ml (yes/no), ART commenced in the first year of life (yes/no), ART commenced in the first 2 years of life (yes/no), nadir CD4% and proportion of life with viral load < 50 c/ml expressed as a percentage. HIV^+^ children had historical data on three to four monthly measures of VL. The proportion of life with an undetectable VL was estimated assuming that, prior to commencing ART, the VL was always detectable. A significance level of *P < *0·05 was considered significant.

## Results

Thirty HIV^−^ and 58 HIV^+^ children were recruited. The baseline characteristics are summarized in Table 1. In line with the study design and target population, there were significant differences in ethnicity. HIV^−^ median age was lower than HIV^+^ (107·5 months *versus* 151·5 months, *P = *0·009); 44 of 56 (78·6%) of HIV^+^ patients were on ART with an undetectable VL at the time of blood sampling. Of those with detectable VL, two patients had never started ART; the remaining children were on structured treatment interruption or were known to have variable adherence. No child with detectable VL was on ART with known viral resistance to their current regimen. All ART‐treated children were on standard three‐drug therapy consisting of a non‐nucleoside reverse transcriptase inhibitor or a protease inhibitor plus two nucleoside/nucleotide reverse transcriptase inhibitors. Median VL was 1227 c/ml [interquartile range (IQR) = 368–14 236] in those with detectable HIV at the time of blood sampling.

**Table 1:** Baseline characteristics plus lymphocyte, T and B cell subset results

		HIV^−^ (*n* = 30)		HIV^+^ (*n* = 56)	
Sex	Female	14	(46·7)	25	(44·6)
	Male	16	(53·3)	31	(55·4)
Ethnicity	White	4	(13·3)	2	(3·6)
	Mixed	0	(0)	3	(5·4)
	Asian	0	(0)	3	(5·4)
	Black Caribbean	4	(13·3)	2	(3·6)
	Black African	13	(43·3)	45	(80·4)^a^
	Other	9	(30)	1	(1·8)
	Age/months	107·5	(19–195)	151·5^b^	(12–209)
	Nadir CD4%	–	–	13	(0–48)
	Proportion of life VL<50 copies/ml	–	–	0·47	(0–0·98)
	VL < 50 copies/ml	–	–	44	(78·6)
	ART commenced in 1st year	–	–	10	(17·9)
	ART commenced in 1st 2 years	–	–	15	(26·8)
Lymphocyte subsets	Lymph cells/μl	2250·3	(1892·2–2676·2)	2419·11^c^	(2145·4–2727·8)
	CD3 cells/μl	1631·2	(1375·7–1934·1)	1839·4^c^	(1632·4–2072·8)
	CD3%	71·1	(68·0–74·4)	73·8	(71·7–75·8)
	CD4 cells/μl	915·7	(775·3–1081·5)	799·1^§^	(674·8–946·3)
	CD4%	40·1	(37·0–43·6)	32·0^c^	(29·0–35·3)
	CD8 cells/μl	558·1	(451·7–689·6)	849·6§¶	(748·3–964·6)
	CD8%	24·5	(22·7–26·3)	34·0^c^	(31·1–37·1)
	CD19 cells/μl	422·0	(323·9–549·7)	392·1§	(329·4–466·8)
	CD19%	18·3	(15·9–21·2)	16·4§	(14·9–18·0)
	CD56 cells/μl	111·1	(80·7–152·9)	109^c^	(88·1–134·7)
	CD56%	5·0	(3·8–6·5)	4·4	(3·6–5·2)
CD19^+^ B cell subsets					
Transitional	CD10^+^CD21^lo^CD27^–^%	1·20	(0·86–1·66)	1·34	(1·09–1·65)
Plasmablasts	CD10^−^CD27^++^IgD−%	0·88	(0·66–1·16)	0·84	(0·63–1·10)
Naive mature	CD27^−^CD21^+^%	71·83	(68·40–75·42)	73·96	(69·91–78·25)
Resting memory	CD27^+^CD21^+^%	18·77	(15·95–22·09)	12·62^d^	(10·78–14·78)
Activated memory	CD27^+^CD21^−^%	2·06	(1·62–2·64)	1·89	(1·46–2·46)
Exhausted/tissue‐like memory	CD27^−^CD21^−^%	3·76	(2·99–4·72)	5·06	(4·13–6·20)
Naive	CD27^−^IgD^+^%	70·38	(66·55–74·44)	75·44^d^	(72·78–78·20)
IgD^+^ memory	CD27^+^IgD^+^%	9·82	(8·24–11·71)	6·60^d^	(5·66–7·68)
Class‐switched memory	CD27^+^IgD^−^%	11·31	(9·46–13·52)	8·71^d^, ^e^	(7·34–10·34)
Double negative	CD27^−^IgD^−^%	4·69	(3·86–5·70)	5·26	(4·51–6·12)
CD4^+^ T cell subsets					
Memory	CD45RO^+^%	32·05	(28·38–36·20)	34·10	(31·01–37·51)
Tfh‐ like	CD45RO^+^CXCR5^+^%	26·26	(23·69–29·10)	17·36^¶^	(13·93–21·64)
ICOS^+^ Tfh‐like	CD45RO^+^CXCR5^+^ICOS^+^%	0·75	(0·53–1·07	0·59	(0·43–0·82)

Sex, ethnicity, HIV viral load status and highly active retroviral therapy (HAART) commenced in the first year of life, HAART commenced in the first 2 years of life are presented as number and percentage. Age, nadir CD4% and proportion of life with undetectable viral load are presented as median and range. Lymphocyte, T and B cell subsets are presented as geometric mean and 95% confidence interval. CD10^+^CD21^lo^CD27^−^ (transitional B cells) and CD10^–^CD27^++^imunoglobulin (Ig)D^–^ (plasmablasts) reported as a proportion of CD19^+^ cells. Remaining B cell subsets reported as a proportion of CD19^+^ after exclusion of transitional B cells and plasmablasts. CD45RO^+^ reported as a proportion of CD4^+^. CD45RO^+^CXCR5^+^ (Tfh‐like cells) and CD45RO^+^CXCR5^+^ICOS^+^ reported as a proportion of CD4^+^CD45RO^+^. HIV− = HIV‐uninfected child healthy control; HIV^+^ = HIV‐infected child; VL = HIV viral load; ART = anti‐retroviral therapy; ICOS = inducible T cell co‐stimulator.

a Proportion significantly different: HIV^+^ *versus* HIV^−^ (*P < *0·005).

b Median significantly different from HIV^–^ (*P < *0·05).

c Significant age × group interaction when comparing HIV^–^ *versus* HIV^+^ (*P < *0·05). §Significant independent age effect when comparing HIV^–^ *versus* HIV^+^ (*P < *0·05).

d Significant independent group effect when comparing HIV^–^ *versus* HIV^+^ (*P < *0·05).

e Non‐significant after adjusting for detectable viraemia (VL > 50 copies/ml) (*P *> 0·05).

We first compared LSS and B and T cell subsets between HIV^+^ and HIV^−^ children and adolescents (Table 1, Fig. 1e, Supporting information, Figs S1 and S2)

### Lymphocyte subsets

There were significant differences in LSS between groups, the pattern of difference often varying with age (interaction effect). Regression plots for lymphocyte subset number and percentage in relation to age are shown in the Supporting information, Fig. S1. There were significant interactions between patient group and age for lymphocyte, CD3^+^, CD4^+^ and CD56^+^ cell counts, and CD8 and CD4 percentage (*P < *0·05). Lymphocyte and CD3^+^ counts for younger children were higher for HIV^+^ than HIV^−^, but more equivalent in older children. For CD4^+^ count, younger HIV^+^ children had higher counts than HIV^−^ children and older HIV^+^ children had lower counts than HIV^−^. For CD56^+^ count, older children in the HIV^+^ group had lower counts than HIV^−^ children of a similar age. For CD8%, older HIV^+^ children had higher percentages than HIV^−^ children of a similar age. Conversely, older HIV^+^ children had lower CD4% than HIV^−^ children of a similar age. The only subset for which there was a significant independent group effect after adjusting for age was CD8^+^ count, with higher CD8^+^ counts in the HIV^+^ group than in the HIV^−^ group. There was a significant independent age effect for CD19^+^ count and percentage (older children having lower counts and percentage). All effects remained significant after adjusting for detectable viraemia.

### Follicular T helper (Tfh)‐like cells

After adjusting for age, CD45RO^+^CXCR5^+^ (Tfh‐like) percentages were lower in HIV^+^ than in HIV^−^ children (*P < *0·05) (Table 1, Fig. 1e). This remained significant after correcting for detectable viraemia. There was no difference in proportions of CD4^+^CD45RO^+^ T cells or ICOS^+^ Tfh‐like cells.

### B cell subsets

There were significant differences in B cell subset percentages in children with perinatally acquired HIV when compared to healthy children (Table 1, Fig. 1e). Representative contour plots for gating CD27 *versus* CD21 and CD27 *versus* IgD are shown in Fig. 1c. Regression plots for those subsets for which there was a significant difference between groups are shown in Fig. 1e (for remaining subsets see Supporting information, Fig. S2a). No significant interaction effects were observed. After adjusting for age, resting memory B cell percentages were lower in HIV^+^ than HIV^−^ (*P < *0·005). This difference was also seen in both IgD^+^ memory (*P < *0·005) and class‐switched memory B cell subsets (*P* < 0·05). Naive B cell proportions were higher in HIV^+^ than HIV^−^ (*P < *0·05). After adjustment for detectable viraemia (VL > 50 c/ml), there was no significant difference in class‐switched memory B cells.

We next analysed data from HIV^+^ children alone to investigate the relationship between HIV treatment history and other clinical parameters and lymphocyte, B and T cell subsets (Supporting information, Table S1).

### Lymphocyte subsets

After adjusting for age, detectable viral load was associated with significantly lower CD4^+^ and CD56^+^ cell counts (*P < *0·0001 and *P = *0.021, respectively) and percentages (*P < *0·0001 and *P = *0.005, respectively) and higher CD8^+^ counts (*P = *0·002) and percentages (*P < *0·0001). A larger proportion of life with undetectable viral load was associated with higher CD4^+^ counts (*P = *0·001) and percentages (*P < *0·0001) and lower CD8^+^ counts (*P = *0·004) and percentages (*P < *0·0001), having adjusted for age. After adjusting for detectable HIV viraemia, only a higher CD4 percentage was associated significantly with a larger proportion of life spent with undetectable viral load. HIV treatment in the first year of life was also found to be associated with higher CD4 percentage after adjusting for age and detectable viraemia (*P = *0·007). There was no association of nadir CD4% or treatment in the first 2 years of life with any lymphocyte subset after adjusting for age and detectable viraemia.

### Tfh‐like cells

After adjusting for age, a larger proportion of life spent with undetectable viral load was associated with lower percentages of CD4^+^CD45RO^+^ T cells (*P = *0·026). In addition, treatment commenced in the first year of life was associated with lower CD4^+^CD45RO^+^ cell percentages (*P = *0·016). These associations remained significant after correcting for detectable viraemia. No association was found between Tfh‐like cells and the clinical variables assessed, including viral load > 50 c/ml, ART commenced in the first year of life, ART commenced in the first 2 years of life, nadir CD4% and proportion of life with viral load < 50 c/ml.

### B cell subsets

Alteration in B cell subsets was more pronounced in HIV viraemic children and were also associated with a larger proportion of life spent with detectable viral load. After adjusting for age, children with a detectable VL had higher percentages of activated and exhausted/tissue‐like memory B cells (*P = *0·003 and *P < *0·0001, respectively) and correspondingly lower percentages of resting memory and naive B cells (*P = *0·001 and *P = *0·025, respectively). Lower percentages of class‐switched memory (*P = *0·048) and higher transitional B cell percentages (*P = *0·03) were also observed. A larger proportion of life spent with undetectable viral load was associated with a higher proportion of resting memory, IgD^+^ memory and class‐switched memory B cells (*P < *0·0001, *P = *0·014 and *P = *0·001, respectively). These associations remained significant after adjusting for detectable viraemia. Reduced exhausted/tissue‐like memory B cells were also associated with a larger proportion of life spent with undetectable VL (*P = *0·002); however, this was non‐significant after adjusting for detectable viraemia. No association was found between any B cell subset, treatment commenced in the first 1 or 2 years of life or nadir CD4%.

Lastly, we investigated the relationship between B and T cell subsets.

After adjustment for age there were significant positive associations between CD4^+^ T cell percentage and resting memory [regression coefficient = 0·957, 95% confidence interval (CI) = 0·564–1·350, *P < *0·001], IgD^+^ memory (regression coefficient = 0·478, 95% CI = 0·042–0·915, *P = *0.032) and class‐switched (regression coefficient = 0·791, 95% CI = 0·329–1·254, *P = *0·001) memory B cell percentages. A significant negative association between CD4^+^ percentage and exhausted/tissue‐like (regression coefficient = −0·903, 95% CI = −1·459–0·348, *P = *0·002) was also found. After correcting for detectable viraemia, the association with exhausted/tissue‐like memory B cells was not significant. At any given age a higher CD4^+^ T cell percentage in children with perinatally acquired HIV is associated with a higher percentage of resting, IgD^+^ and class‐switched memory B cells. No significant association was found between memory T cell, Tfh‐like and ICOS^+^ Tfh‐like and B cell subsets.

## Discussion

To our knowledge, this is the first study to report both circulating B cell subsets and Tfh‐like cells in children with perinatally acquired HIV compared to healthy children, adding to existing published data on B cell phenotypes in HIV‐infected children. We have shown perinatally acquired HIV infection to be associated with reduced Tfh‐like cells and resting memory and IgD^+^ memory B cells. Detectable viraemia is associated with reduced resting and class‐switched memory B cell percentages and increased activated and exhausted/tissue‐like memory B cell percentages. Alterations in B cell subsets exist despite suppressive ART; however, a greater proportion of life spent on ART is associated with a more preserved B cell phenotype.

Our observed differences in LSS are consistent with the published data on incomplete immune reconstitution in ART‐treated children. Despite suppressive ART, some children continue to have lower CD4 counts (reviewed in 13) and CD8 cell counts remain elevated even when VL is undetectable 14. Our finding that a greater proportion of life spent on suppressive ART and ART commencement in the first year of life are associated with higher CD4 counts/percentages is consistent with accumulating data that earlier and sustained VL suppression may be associated with CD4 count preservation 13, 15.

In parallel, we have found perinatally acquired HIV to be associated with decreased resting memory and IgD^+^ memory B cell percentages, while a greater proportion of life spent on suppressive ART is associated with higher memory B cell percentages. Furthermore, we found a significant positive association between CD4 percentage and resting memory, IgD^+^ and IgD^−^ memory B cells. This is consistent with existing data sugggesting that earlier ART commencement may also preserve the B cell compartment 2, 5, 13. Taken together, our data support recent changes to guidelines recommending earlier ART initiation at higher CD4 thresholds in childhood 16. Mechanisms contributing to sustained B cell phenotypical abnormalities on ART are unclear; however, it is considered to be due probably to a combination of irreversible damage to lymphoid tissue architecture and function (sustained during viraemic periods) and ongoing low‐level immune activation 5. Double‐negative B cells (CD27^−^IgD^−^), similar to a population observed in elderly people, have been shown recently to be increased in children with chronic HIV infection, a phenomenon that may be related to ‘immune senescence’. We did not observe this, due possibly to our cohort being younger or there being a lower proportion of viraemic patients than in the published reports 17, 18.

Deficiencies in memory B cell populations, at least those post‐germinal centre, could theoretically be related to the reduction in Tfh‐like cells we observed. Lower proportions of dysfunctional circulating CD4^+^CXCR5^+^ populations have been reported recently in ART‐naive adult HIV infection, with proportions increasing following ART initiation 19. As is the case for our cohort, this may reflect a true reduction in cell numbers, although recent work suggests that it is due more probably to a redistribution of cells within lymphoid tissue 20 (reviewed in 7). Again, it is important to note that differences in our findings and published work to date are likely to exist in view of the rapidly changing cell dynamics of the child's developing immune system compared to adults. Tfh cell responses to immunization have been shown recently to be relatively impaired in neonatal mice when compared to adult mice 21. It remains to be shown whether the same is true in human infants.

Whether or not related, the fact that deficits in both Tfh‐like cells and memory B cells exist in ART‐treated children with perinatally acquired HIV, and that viraemic children have higher numbers of tissue‐like/exhausted and activated memory B cells, is cause for concern. It has implications for ART treatment strategies, as earlier and sustained ART may preserve T and B cell phenotypes, and for vaccination practices, where serological response may be optimized by immunizing once established on fully suppressive ART. This is relevant for both routine immunizations in HIV‐infected children and also for therapeutic HIV vaccination as part of a future potential combined approach to HIV treatment.

Our study is limited by a lack of matching for ethnicity between the HIV^+^ and HIV^−^ groups. This was unavoidable, owing to the time available for recruitment and demographics of the population from which healthy children were recruited. Better matching for ethnicity would have helped to exclude this as a potential confounding factor. However, at the time of writing we are not aware of any existing published data reporting variations in Tfh‐like cells or memory B cell populations between ethnic groups. Furthermore, the median age of the HIV^+^ group was higher than that of the HIV^−^ group. This has been addressed by adjusting for age during statistical analysis. The need for this would have been avoided through precise age‐matching at the time of recruitment, but this was not possible. There have been rapid recent developments in the phenotypical characterization of both memory B and Tfh‐like cells (summarized in 5). The addition of further markers/additional gating strategies and functional analysis of Tfh‐like cells, although adding to the complexity of the data analysis, would have enhanced our findings and will be the focus of future work. The implications of the observed abnormalities for vaccine responses has also been assessed, and is the subject of a separate paper 22.

In summary, findings from our work and other studies of human HIV infection and non‐human primate SIV models are indicative of a complex interplay between HIV, Tfh and B cells in lymphoid tissue and peripheral circulation to the detriment of humoral immunity, which is only partially corrected by ART. Current evidence suggests there may be an accumulation of HIV‐specific and/or infected Tfh in lymph nodes, which correlates with the altered lymph node B cell phenotype. This is reflected in altered T and B cell phenotypes in the peripheral blood which, in turn, can be correlated with impaired response to vaccination 5. Our results not only emphasize that T and B cell abnormalities persist in children with HIV despite suppressive ART, but also provide evidence that earlier and sustained ART serves to maximize the potential for normal immune development beyond its effect on CD4^+^ T cells.

## Disclosure

The authors declare no conflicts of interest relating to this work.

## Author contributions

A. B. planned and completed the project under the supervision of B. K., P. K. and D. G. A. B. applied for funding and ethical approval. A. B. recruited all patients and performed all laboratory work under the supervision of M. H. and P. K. H. L. provided clinical collaboration. A. B. performed all statistical analysis and prepared the manuscript. All authors have reviewed and approved the final manuscript.

## Supporting information


**Fig. S1**. Regression plots comparing lymphocyte subsets in healthy children with children with perinatally acquired HIV. Significant interactions between age and group were present for lymphocyte, CD3^+^, CD4^+^ and CD56^+^ cell counts (*P < *0·05) and for CD4^+^ percentage (*P < *0·005). Significant independent age effects were present for CD8^+^ and CD19^+^ cell counts and CD19^+^ percentage (*P < *0·005). Significant independent group effect was present for CD8^+^ cell count (*P < *0·005). HIV^−^ = HIV‐uninfected child healthy control; HIV^+^ = HIV‐infected child; ln = natural log, cell counts measured in cells/μl.

**Fig. S2**. Regression plots comparing (a) B cell and (b) T cell subsets for which there was no significant group effect when comparing healthy children with children with perinatally acquired HIV. HIV^−^ = HIV=uninfected child healthy control; HIV^+^ = child patient; ln = natural log. Percentages are reported as described in Table 1.

**Table S1**. Results of separate linear regression analyses for detectable viral load and proportion of life undetectable after adjusting for age for HIV group alone.
