# Supplementary figures and images for: The influence of paediatric HIV infection on circulating B cell subsets and CXCR5+ T helper cells
Source: Clin Exp Immunol. 2015 May 6;181(1):110–7. doi: 10.1111/cei.12618 (PMC4469160; doi:10.1111/cei.12618)

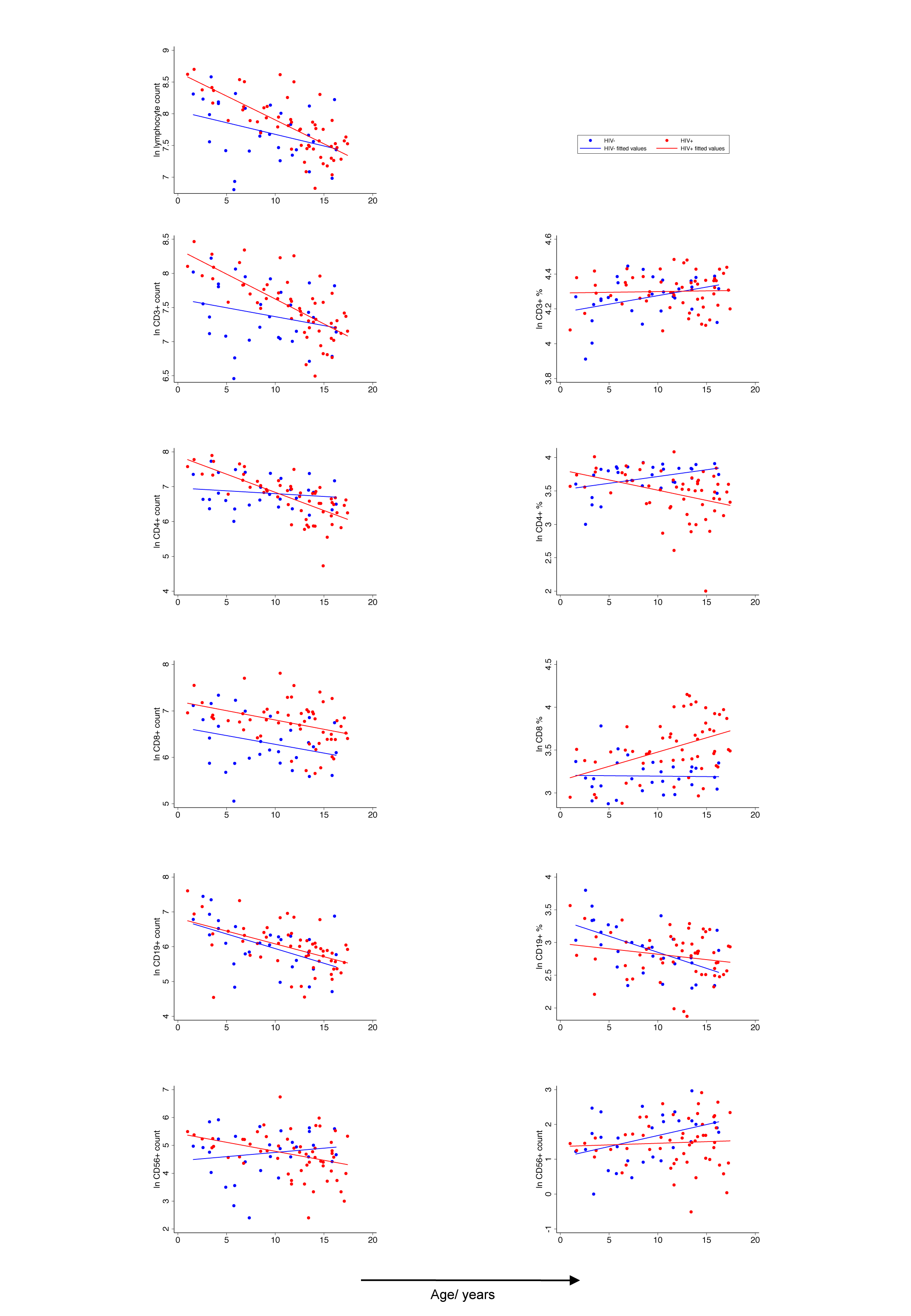

Supplement: Supplementary file 1 — Fig. S1. Regression plots comparing lymphocyte subsets in healthy children with children with perinatally acquired HIV. Significant interactions between age and group were present for lymphocyte, CD3+, CD4+ and CD56+ cell counts (P < 0·05) and for CD4+ percentage (P < 0·005). Significant independent age effects were present for CD8+ and CD19+ cell counts and CD19+ percentage (P < 0·005). Significant independent group effect was present for CD8+ cell count (P < 0·005). HIV− = HIV‐uninfected child healthy control; HIV+ = HIV‐infected child; ln = natural log, cell counts measured in cells/μl. [file CEI-181-110-s001.tif]

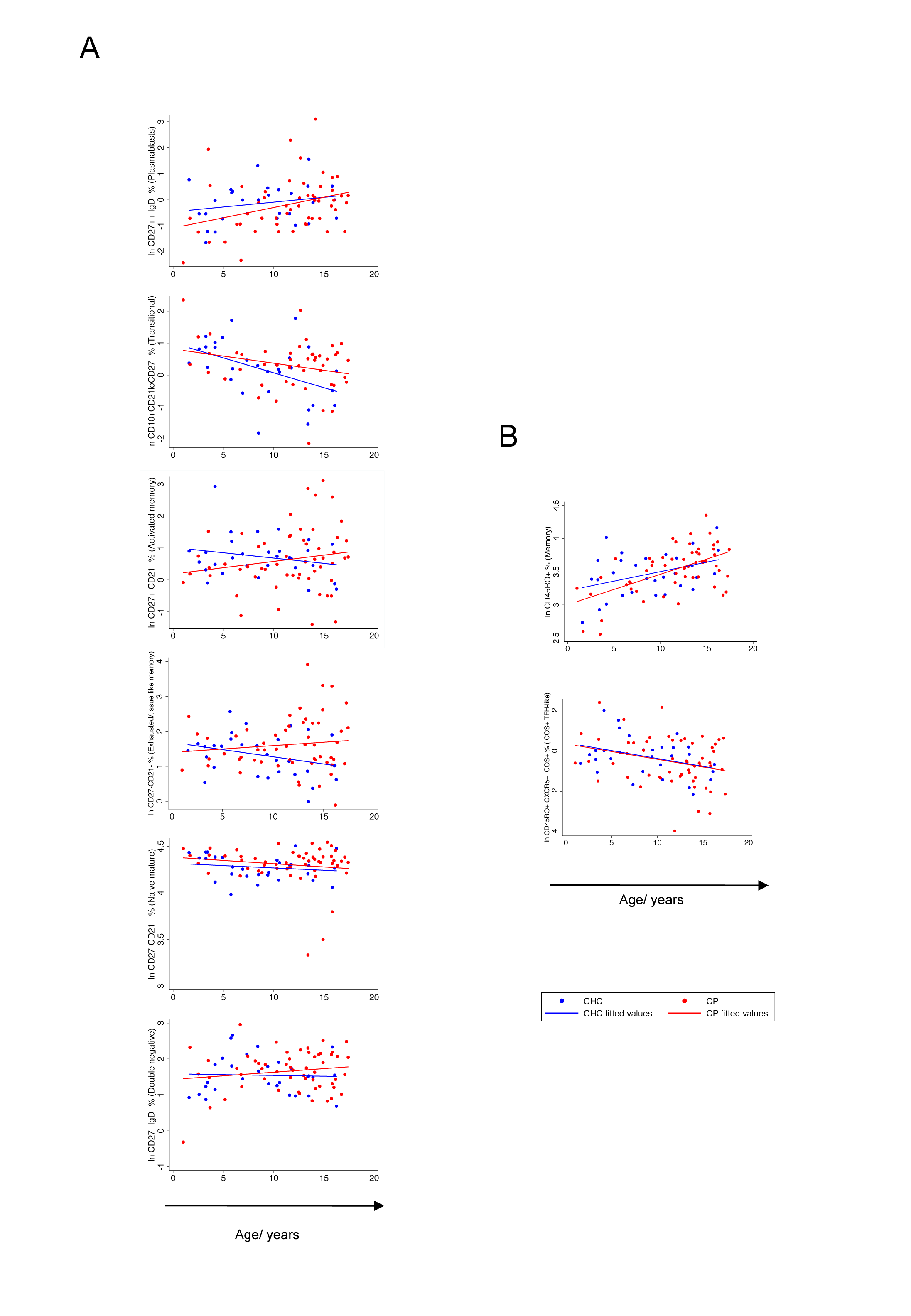

Supplement: Supplementary file 2 — Fig. S2. Regression plots comparing (a) B cell and (b) T cell subsets for which there was no significant group effect when comparing healthy children with children with perinatally acquired HIV. HIV− = HIV=uninfected child healthy control; HIV+ = child patient; ln = natural log. Percentages are reported as described in Table 1. [file CEI-181-110-s002.tif]
