# Supplementary material for: The influence of paediatric HIV infection on circulating B cell subsets and CXCR5+ T helper cells
Source: Clin Exp Immunol. 2015 May 6;181(1):110–7. doi: 10.1111/cei.12618 (PMC4469160; doi:10.1111/cei.12618)
Supplement: Supplementary file 3 — Table S1. Results of separate linear regression analyses for detectable viral load and proportion of life undetectable after adjusting for age for HIV group alone. [file CEI-181-110-s003.docx]

Supplementary Table 1. Results of separate linear regression analyses for detectable viral load and proportion of life undetectable after adjusting for age for HIV+ group alone. Antilog of regression coefficients and 95% confidence intervals and corresponding *p* values are reported. B and T-cell subsets proportions are reported as described in Table 1. (abs = absolute cell count in cells/μl, VL = viral load, coef= regression coefficient, CI= confidence interval, NS = non significant (*p*>0.05))

^a^ Non significant after adjusting for detectable viraemia (VL>50 copies/ml)

|  |  | VL detectable (yes/no) | | | | Percentage of life VL undetectable | | | |
| --- | --- | --- | --- | --- | --- | --- | --- | --- | --- |
|  |  | antilog coef | antilog 95% CI | | *p* | antilog coef | antilog 95% CI | | *p* |
| **Lymphocyte subsets** |  |  |  |  |  |  |  |  |  |
|  | CD3 cells/μl | 0.964 | 0.781 | - 1.119 | NS | 1.000 | 0.997 | - 1.003 | NS |
|  | CD3% | 1.009 | 0.941 | - 1.082 | NS | 1.001 | 1.000 | - 1.002 | NS |
|  | CD4 cells/μl | 1.696 | 1.312 | - 2.191 | <0.0005 | 1.007 | 1.003 | - 1.012 | <0.005 |
|  | CD4% | 1.765 | 1.489 | - 2.092 | <0.0005 | 1.008 | 1.005 | - 1.011 | <0.0005 ^a^ |
|  | CD8 cells/μl | 0.644 | 0.491 | - 0.845 | <0.005 | 0.994 | 0.989 | - 0.998 | 0.0040 |
|  | CD8% | 0.668 | 0.565 | - 0.789 | <0.0005 | 0.995 | 0.992 | - 0.997 | <0.0005 |
|  | CD19 cells/μl | 0.959 | 0.654 | - 1.405 | NS | 0.996 | 0.991 | - 1.002 | NS |
|  | CD19% | 1.033 | 0.816 | - 1.307 | NS | 0.997 | 0.993 | - 1.001 | NS |
|  | CD56 cells/μl | 1.757 | 1.090 | - 2.830 | <0.05 | 1.005 | 0.997 | - 1.012 | NS |
|  | CD56% | 1.854 | 1.210 | - 2.840 | <0.005 | 1.006 | 0.999 | - 1.013 | NS |
| **B-cell subsets** |  |  |  |  |  |  |  |  |  |
| *Transitional* | CD10+CD21^lo^CD27- % | 0.584 | 0.360 | - 0.948 | <0.05 | 1.000 | 0.992 | 1.008 | NS |
| *Plasmablasts* | CD10-CD27++IgD- % | 0.972 | 0.501 | - 1.89 | NS | 1.006 | 0.995 | 1.016 | NS |
| *Naive mature* | CD27-CD21+% | 1.167 | 1.020 | - 1.335 | <0.05 | 1.001 | 0.999 | 1.003 | NS |
| *Resting memory* | CD27+CD21+% | 1.863 | 1.302 | - 2.666 | <0.005 | 1.013 | 1.008 | 1.018 | <0.0005 |
| *Activated memory* | CD27+CD21-% | 0.399 | 0.220 | - 0.722 | <0.005 | 0.997 | 0.987 | 1.007 | NS |
| *Exhausted/tissue like memory* | CD27-CD21-% | 0.406 | 0.260 | - 0.634 | <0.0005 | 0.988 | 0.981 | 0.996 | <0.005 ^a^ |
| *Naive* | CD27-IgD+ % | 0.981 | 0.896 | - 1.074 | NS | 0.999 | 0.997 | 1.000 | NS |
| *IgD+ memory* | CD27+IgD+ % | 1.039 | 0.709 | - 1.523 | NS | 1.007 | 1.002 | 1.013 | <0.05 |
| *Class-switched memory* | CD27+IgD- % | 1.517 | 1.003 | - 2.294 | <0.05 | 1.011 | 1.005 | 1.017 | <0.005 |
| *Double negative* | CD27-IgD- % | 1.15 | 0.788 | - 1.680 | NS | 1.002 | 0.996 | 1.008 | NS |
| **T-cell subsets** |  |  |  |  |  |  |  |  |  |
| *Memory* | CD45RO^+^ % | 0.916 | 0.752 | - 1.115 | NS | 0.997 | 0.994 | 0.999 | <0.05 |
| *T_FH_- like* | CXCR5^+^ % | 0.959 | 0.553 | - 1.661 | NS | 0.998 | 0.990 | 1.007 | NS |
| *ICOS+ T_FH_- like* | CXCR5^+^ ICOS^+^ % | 0.539 | 0.251 | - 1.158 | NS | 0.993 | 0.981 | 1.005 | NS |
